# Editorial – Current capacities and future possibilities of large language models in orthopaedic surgery

**DOI:** 10.1002/jeo2.70273

**Published:** 2025-05-26

**Authors:** Assil Mahamid, Lior Laver, Sana Zahalka, Felix Oettl, Eyal Behrbalk, Michael T. Hirschmann, Kristian Samuelsson

**Affiliations:** ^1^ Department of Orthopedics Hillel Yaffe Medical Center Hadera Israel; ^2^ Technion Israel Institute of Technology Haifa Israel; ^3^ Joyce and Irving Goldman Medical School, Faculty of Health Sciences Ben‐Gurion University of the Negev Beer‐Sheva Israel; ^4^ Departments of Hip and Knee Replacement Hospital for Special Surgery New York New York USA; ^5^ Department of Orthopedic Surgery and Traumatology Kantonsspital Baselland Bruderholz Switzerland; ^6^ Department of Orthopaedics, Institute of Clinical Sciences, Sahlgrenska Academy University of Gothenburg Gothenburg Sweden

**Abbreviations**
AI: artificial intelligence
LLM: large language model
USMLE: United States Medical Licensing Examination
WAME: World Association of Medical Editors

The accelerated advancement of artificial intelligence (AI) and large language models (LLMs) like GPT‐4 has paved the way for revolutionary shifts in almost all medical specialties. Orthopaedic surgery, traditionally characterized by its reliance on physical and radiographic diagnosis as well as surgical expertise, is increasingly integrating these advanced AI technologies into clinical practice. This editorial evaluates the use of LLMs in orthopaedic surgery, the influence that prominent LLMs have had on the field, and the potential these technologies hold to improve patient care and medical research in the future. Figure 1 provides a concise explanation of the operational mechanism of the LLM.

**Figure 1:**
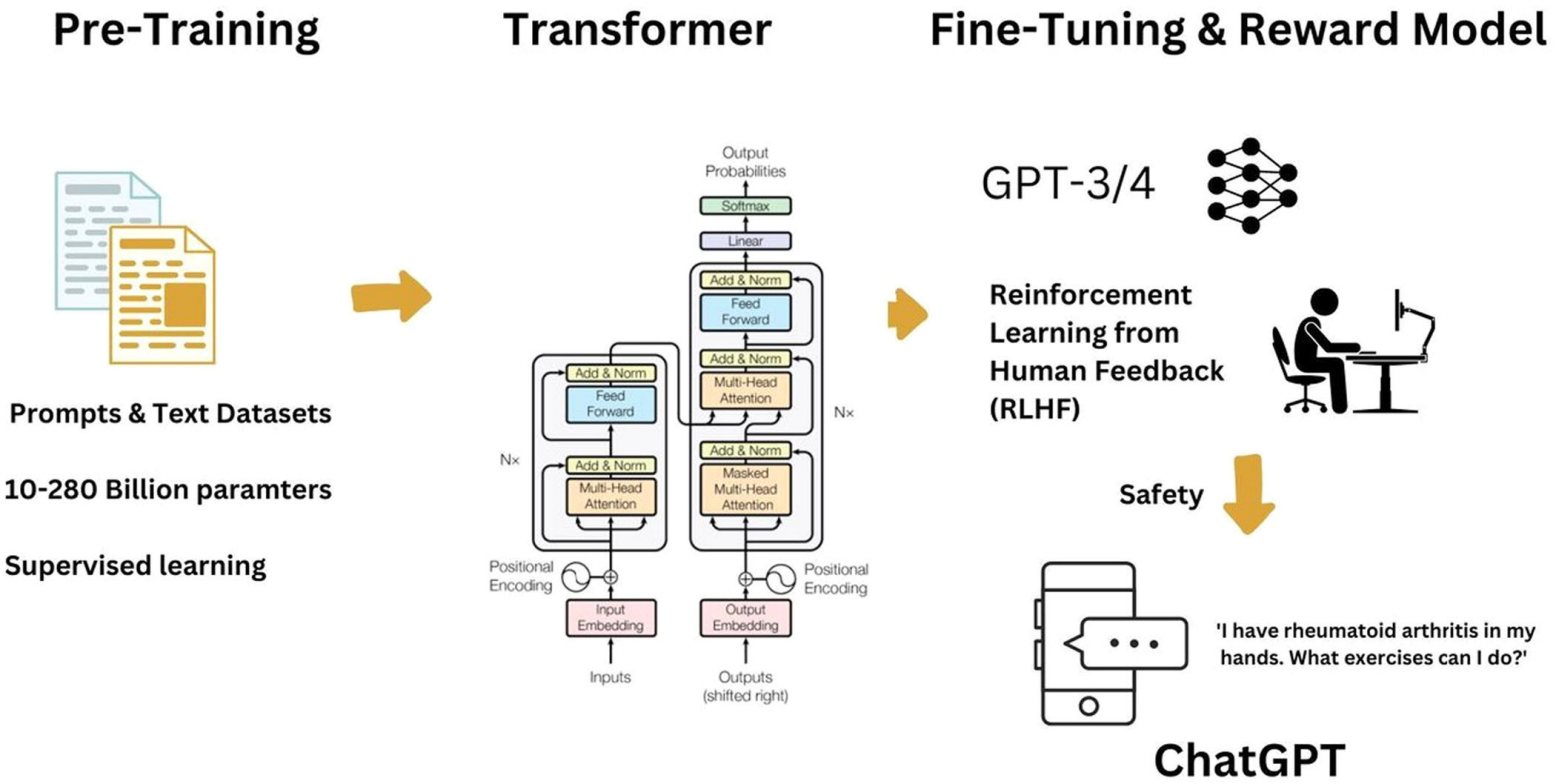
Words are represented as numerical vectors (word embeddings), capturing semantic relationships. An encoder translates words into embeddings, while a decoder reconstructs meaningful text. Advancements from earlier models involved reinforcement learning with human feedback, refining responses for accuracy and coherence while incorporating safeguards to prevent harmful content [1].

## CURRENT IMPLEMENTATION OF LLMS IN ORTHOPAEDIC SURGERY

There has been an increasing interest in using LLMs in the medical field in general and orthopaedic surgery in particular. The potential use of LLMs in orthopaedic surgery has been vast and variable in fields in clinical use, research conduction, medical education, as well as patient education.

Recent studies show that AI and LLMs can aid in creating clinical letters and care strategies for typical orthopaedic scenarios in clinical practice. Large volumes of unstructured data, including imaging reports, surgical notes and patient records, are processed by these models, offering a more thorough understanding of patient conditions and generating understandable, efficient and generally accurate texts. However, occasionally, the omitted output may be inconsistent, lack key details or offer general recommendations [2, 3].

Interestingly, LLMs can offer treatment recommendations based on clinical data, such as magnetic resonance imaging reports. Still, their utility is limited by a need for further context and specificity, necessitating oversight by healthcare professionals [4].

Furthermore, LLMs can assist clinicians as early on in their careers as medical school and residency, as several noteworthy studies have demonstrated that LLMs meet the passing criteria for both the United States Medical Licensing Examination (USMLE) and the Orthopaedic board examinations [5, 6, 7, 8].

Apart from clinicians, LLMs may deliver clear and concise answers to frequently asked patient inquiries, providing a dependable knowledge source for prevalent orthopaedic issues. Those models can condense complex facts into comprehensible summaries, which are useful for patients aiming to understand their conditions and therapy alternatives, subsequently referring them to the appropriate specialist, potentially cutting back unwarranted appointments and alleviating the workload of general physicians [3, 9, 10].

## LEADING PLATFORMS AND THEIR IMPACT ON ORTHOPAEDIC SURGERY

Several LLM platforms have emerged as leaders in this domain, each bringing unique strengths to orthopaedic practice. OpenAI's GPT‐4, and Anthropic's Claude AI are among the most notable. These platforms are designed to understand and generate human‐like text, making them invaluable tools for both clinical and research applications.

*GPT‐4o*, has shown potential in numerous aspects of orthopaedic practice. In clinical decision‐making, it can assist orthopaedic surgeons by providing quick access to relevant medical information, offering diagnostic suggestions and aiding in treatment planning [11]. In orthopaedic research, the AI model can assist in conducting literature reviews, draft research proposals and manuscripts, as well as help analyze research data [4, 12]. Moreover, when given the Orthopaedic In‐Service Training Exam (OITE) as input, ChatGPT performed around the first postgraduate year level, providing a consistently logical rationale for most of the correct answers [13]. Similar results were observed with the American Board of Orthopaedic Surgery exams [5, 6, 7]. This emphasizes the model's potential in providing interactive learning experiences for medical students and residents.

*Claude AI* is another platform gaining attention due to its potential medical applications, particularly in orthopaedic surgery. A cross‐sectional study assesses the effectiveness of LLMs in responding to surgery‐related patient inquiries, focusing on accuracy, relevance, clarity and emotional sensitivity. The results indicate that LLMs perform well in these areas, with Claude surpassing ChatGPT and Google's Bard [14].

*Gemini*, developed by Google, is an advanced AI tool engineered to generate human‐like responses by processing extensive data sets. In paediatric orthopaedics, the emerging role of LLMs, such as Gemini, in aiding clinical decision‐making and patient education is gaining increasing attention [15, 16]. In comparison to evidence‐based guidelines on supra‐condylar and diaphyseal femur fractures, such as those provided by the American Academy of Orthopaedic Surgeons, Gemini exhibited an accuracy rate similar to that of other LLMs, including ChatGPT‐4.0, in delivering treatment recommendations for common paediatric fractures. However, notable discrepancies were observed in the citation of studies, and instances of overgeneralization in management plans were present, with many responses necessitating substantial modifications [15].

*Bidirectional Encoder Representations from Transformers (BERT)*, while not a chatbot platform, is a prominent neural network‐based method for language processing. It has proven particularly valuable for natural language processing tasks in orthopaedic surgery. The recent launch of the Japanese Orthopaedic Association National Registry (JOANR) demonstrated that BERT significantly outperformed the other methods in accurately identifying key surgical details from operative records, such as the surgical approach and fixation technique. These findings suggest that BERT is the most suitable method for automating data extraction for JOANR, thereby streamlining the registration process and reducing the workload on surgeons [17].

An additional promising feature of BERT was observed in a proof‐of‐concept study demonstrating improved prediction accuracy for surgical case duration, based on BERT's ability to extract features from unstructured clinical data of patients [18], thereby reinforcing LLMs' ability to optimize daily clinical planning and function.

### Concerns

Acknowledging the growing concerns surrounding the use of LLMs in medicine is crucial. These concerns stem from LLMs' potential to generate content that may need to meet the rigorous standards required for medical publications, including issues related to the accuracy of medical information, the risk of introducing biases and the challenges of maintaining the confidentiality of patient data. As these technologies become increasingly integrated into medical research and publication, the medical community must remain vigilant in assessing and mitigating the risks associated with their use.

A recent study revealed a growing trend in using LLMs for writing articles submitted to an orthopaedic journal [19, 20]. The deployment of chatbots for content creation poses considerable risks. Generative AI technologies, particularly LLMs, are well‐known for their potential to produce ‘hallucinations’, wherein they generate information that lacks empirical support [21]. Furthermore, due to the nature of LLMs, which formulate responses based on established data patterns, there is a significant risk that their output may closely resemble pre‐existing literature, raising concerns regarding potential plagiarism. The risk of compromising data privacy is also substantial, especially if patient information or sensitive clinical data is inadvertently included in published content.

The World Association of Medical Editors (WAME) has unequivocally stated that chatbots are not eligible to be credited as authors, as they do not possess the capacity to give ‘final approval’ of manuscripts or to understand conflict of interest declarations [22]. Moreover, WAME recommends that any use of AI in manuscript preparation be transparently disclosed and emphasizes that authors are fully accountable for all content produced by chatbots.

These concerns go beyond the field of research and extend to clinical settings; identified limitations of LLMs included partial responses, insufficient contextual relevance and reliance on outdated information [10].

Having said that, these concerns almost solely arise from studies in the literature that observe publicly available generalized LLMs (e.g., ChatGPT). Despite their increasing popularity and availability, scarce research can be found on Health data companies (e.g., LynxCare) that employ healthcare‐specific LLMs and AI models. These companies, firsthand, recognize the needs and concerns that arise in the medical field and thus provide highly specific models that aim to mitigate the limitations of the above‐mentioned platforms.

### Future implementation in orthopaedic surgery

Looking forward, the potential applications of LLMs in orthopaedic surgery are vast and varied. One promising avenue is the development of AI‐powered surgical robots that can assist or even autonomously perform specific procedures. By integrating LLMs with advanced imaging techniques, these robots could identify the optimal surgical approach in real time, adjusting their strategies based on intraoperative findings. Another exciting prospect is the use of LLMs in personalized medicine. By analyzing genomic data alongside patient history, LLMs could help identify patients more likely to benefit from specific interventions, such as biologic treatments or advanced prosthetics [12]. This tailored approach to patient care could significantly improve outcomes, particularly in complex or refractory cases.

Furthermore, LLMs play a crucial role in the education and training of orthopaedic surgeons. Virtual simulations powered by LLMs provide trainees with realistic, case‐based learning experiences, allowing them to hone their skills in a risk‐free environment [23, 24]. These models could also assess surgical competence, provide objective performance feedback and identify improvement areas.

In conclusion, integrating LLM into orthopaedic surgery represents a significant leap forward in the field. While still in its early stages, implementing these models has already begun to enhance clinical outcomes, streamline administrative tasks, as well as improve patient care. As LLM technology continues to evolve, its role in orthopaedic surgery will likely expand, offering new tools and techniques that could revolutionize how orthopaedic care is delivered. The challenge for the orthopaedic community will be to embrace these innovations while ensuring that they are used in a way that complements, rather than replaces, the expertise and judgement of skilled clinicians.

## AUTHOR CONTRIBUTIONS

Conceptualization and methodology: A.M., L.L. and F.O; Software: J.O. and F.O.; Validation: B.Z., F.O., and F.J.; Writing—Original Draft Preparation: A.M. and S.Z.; Writing—Review and Editing: L.L, E.B and M.H.; Supervision: L.L., K.S., and E.B.

## CONFLICT OF INTEREST STATEMENT

Kristian Samuelsson is a Member of the Board of Directors of Getinge AB (publ) and medtech advisor to Carl Bennet AB. The other authors declare no conflicts of interest.

## ETHICS STATEMENT

This study did not require ethical approval as it did not involve human or animal subjects. Patient consent was not required for this study.
